# Bringing Positive Change—Dyslexic Medical Educators' Experiences of Teaching

**DOI:** 10.1111/tct.70562

**Published:** 2026-11-11

**Authors:** Cleone Riad, Alison Ledger

**Affiliations:** ^1^ Leeds Institute of Medical Education University of Leeds Leeds UK

**Keywords:** dyslexia, dyslexic medical educator, neurodiversity, teaching

## Abstract

**Introduction:**

Dyslexic doctors are increasingly represented within the medical workforce, yet little is known about dyslexic doctors as educators. Dyslexic medical educators contribute to workforce diversity and support diverse student populations; however, their experiences and motivations to teach remain under‐explored.

**Methods:**

Using an interpretivist, neurodiversity‐affirmative approach, we conducted a qualitative study involving semi‐structured interviews with five dyslexic medical educators. Data were analysed using reflexive thematic analysis to explore educators' experiences, motivations to teach and perceptions of how their dyslexia influences their teaching practices.

**Results:**

Five themes were developed: (1) navigating dyslexia as a shortcoming; (2) the ‘extra‐time’ identity; (3) exploring and embracing their dyslexic identity; (4) own learning‐to‐learn as a guide; and (5) the power to bring about positive change.

**Discussion:**

Participants described facing stigma, ableist norms and episodes of difficulty, often linked to an enduring deficit‐narrative within medicine. Notwithstanding these experiences, they developed positive dyslexic identities and foregrounded their strengths in understanding and applying inclusive, learner‐centred teaching practices. Participants benefited from role models and allies and, in turn, experienced a responsibility to advocate positively, challenge deficit‐based narratives and improve educational experiences for others. Our findings position dyslexic medical educators as skilled practitioners who can embody and advocate for inclusion when contexts are supportive.

## 1 Introduction

A diverse healthcare workforce is essential for serving a diverse population [1]. Accordingly, a diverse student population is better supported by a diverse educational workforce. Neurodivergent individuals, including dyslexic individuals, are important members of a diversifying health professions education workforce. As numbers of dyslexic medical students increase, the rates of dyslexic doctors and dyslexic medical educators will rise [2]. It is therefore critical we examine and appreciate the experiences, perspectives and contributions of dyslexic medical educators, a topic that is under‐researched and essential in advancing broader diversity goals within the medical and educational workforce.

Dyslexia can be understood in multiple ways. Under the medical model, dyslexia is considered a deficit that requires dyslexic individuals to adapt to succeed [3]. Under the Equality Act (2010), dyslexia is legally recognised as a disability [4]. Organisations have a legal and moral responsibility to provide inclusive and participatory environments through making reasonable adjustments for dyslexic staff and students.

The neurodiversity paradigm foregrounds difference and diversity and challenges disabling environments such that differences can manifest as strengths [5]. Neurodiversity describes the natural variation of neurocognitive functioning across a neurodiverse population, constructed from neurotypical and neurodivergent individuals, including dyslexic individuals [5, 6]. Under this paradigm, individuals are reclaiming their neurodivergence as an integral part of themselves, cultivating a positive identity [7, 8]. Aligning with this, many neurodivergent individuals prefer identity‐first language, that is, ‘dyslexic individual’.

Literature exploring dyslexic medical educators' experiences is scarce, but research into dyslexic students, doctors and teachers provides insight. Dyslexic medical students and doctors display strengths, including creativity, problem solving and strong communication [9, 10]. They are often skilled at masking being dyslexic, which can prevent or delay diagnosis [10], and they describe feeling pressure to cope without meaningful psychological or pastoral support [11]. Lack of inclusion and understanding from colleagues, supervisors and peers breeds shame [11], inferiority [10] and isolation [9]; feelings that negatively impact self‐esteem and can lead to psychological stress and illness [10, 12, 13]. Due to perceived or real stigma and patient safety concerns, healthcare professionals face challenges disclosing a dyslexia diagnosis [14]. Disclosure is often context‐dependent and more challenging for those with marginalised intersecting identities [2].

Education literature suggests dyslexic individuals are often drawn to teaching [15], seeing dyslexia as a powerful educational advantage [16]. Dyslexic teachers have a strong sense of purpose and commonly share their dyslexic identity to help them frame their experiences positively [15, 17].

Although no studies to date have specifically examined the experiences of dyslexic medical educators, isolated accounts suggest that dyslexic doctors perceive themselves as skilled educators [9, 10, 18], motivated by their own experiences to support students and reframe difficulties [18].

The study aims were to explore how dyslexic medical educators perceive being dyslexic influences their teaching practices and what motivates dyslexic doctors to teach.

## 2 Methods

We undertook this research from an interpretivist standpoint [19] to explore and value individual medical educators' experiences and to adopt an active role in shaping the research findings. Cleone (first author) is herself a dyslexic doctor who worked as a clinical teaching fellow while completing her Masters in Clinical Education degree. Alison (second author) was the research supervisor. We have experience in supporting neurodivergent students and developing and applying inclusive education standards.

Using the neurodiversity paradigm as our conceptual framework, we align with the position that dyslexic individuals can be effective educators, and inclusive environments can allow differences to manifest as strengths [5].

## 3 Ethics

Approval by the University of Leeds Medicine Research Ethics Committee.

### 3.1 Participants and Recruitment

We recruited six dyslexic medical educators who taught medical students regularly and self‐reported a formal dyslexia diagnosis. Participants with multiple neurodivergent diagnoses were included to value diversity and avoid diagnostic silos. We chose a small sample to explore experiences in detail and achieve ‘information power’ [20]. We emailed colleagues who met the inclusion criteria or could share recruitment materials with dyslexic educators. We approached known dyslexic educators to reduce the risk that disclosure would limit participation. Cleone was open about being dyslexic when recruiting, and all had met her at least once before the research. To minimise risks of obligation and coercion, participation was voluntary, with withdrawal possible up to 48‐h post‐data collection. One recruit withdrew prior to data collection. Five participants were interviewed.

### 3.2 Data Collection and Analysis

In 2022, Cleone interviewed participants individually using a guide shared with participants in advance. Questions considered participants' roles as a doctor and medical educator, their motivations to teach, challenges, strategies and strengths related to their dyslexia, disclosure and support. Consent procedures and interviews were conducted via University Microsoft Teams and lasted 60 min. Audio‐video interview recordings were stored on University Microsoft OneDrive before transcription and were subsequently deleted. Consent was stored separately from interview data. Participants were assigned pseudonyms.

We employed reflexive thematic analysis [21] to align with our research questions, data and interpretivist stance. To aid data familiarisation and theme generation, Cleone used strategies matching her dyslexic preferences. She sketched, produced visual representations using Canva software, created voice recordings and then undertook more traditional line‐by‐line textual coding. Cleone grouped the codes into initial themes, then in discussion with Alison, developed and refined the themes and determined the story presented in this paper.

### 3.3 Reflexive Strategies

Cleone was interviewed by Alison using the interview guide and kept a reflective diary to explore her positionality, assumptions and reactions throughout the research. During analysis, we experienced tensions between validating participants' challenges and our desire to avoid a deficit perspective. We recognised the potential to privilege experiences that aligned with our own when developing themes. We navigated these tensions by discussing Cleone's interpretations and considering alternative experiences. For example, we acknowledged Cleone's relative privilege as a White heterosexual doctor, from a university‐educated family, when developing recommendations for bringing about positive change.

## 4 Results

We present direct quotes from our constructed themes and examples of Cleone's reflexive practice to align with our interpretivist perspective. We chose identity‐first language to match current guidance [5, 7, 10] and the preferences of both Cleone and our participants. Visual representations are included to demonstrate an analysis preference of a dyslexic researcher (Figure 1).

**FIGURE 1:**
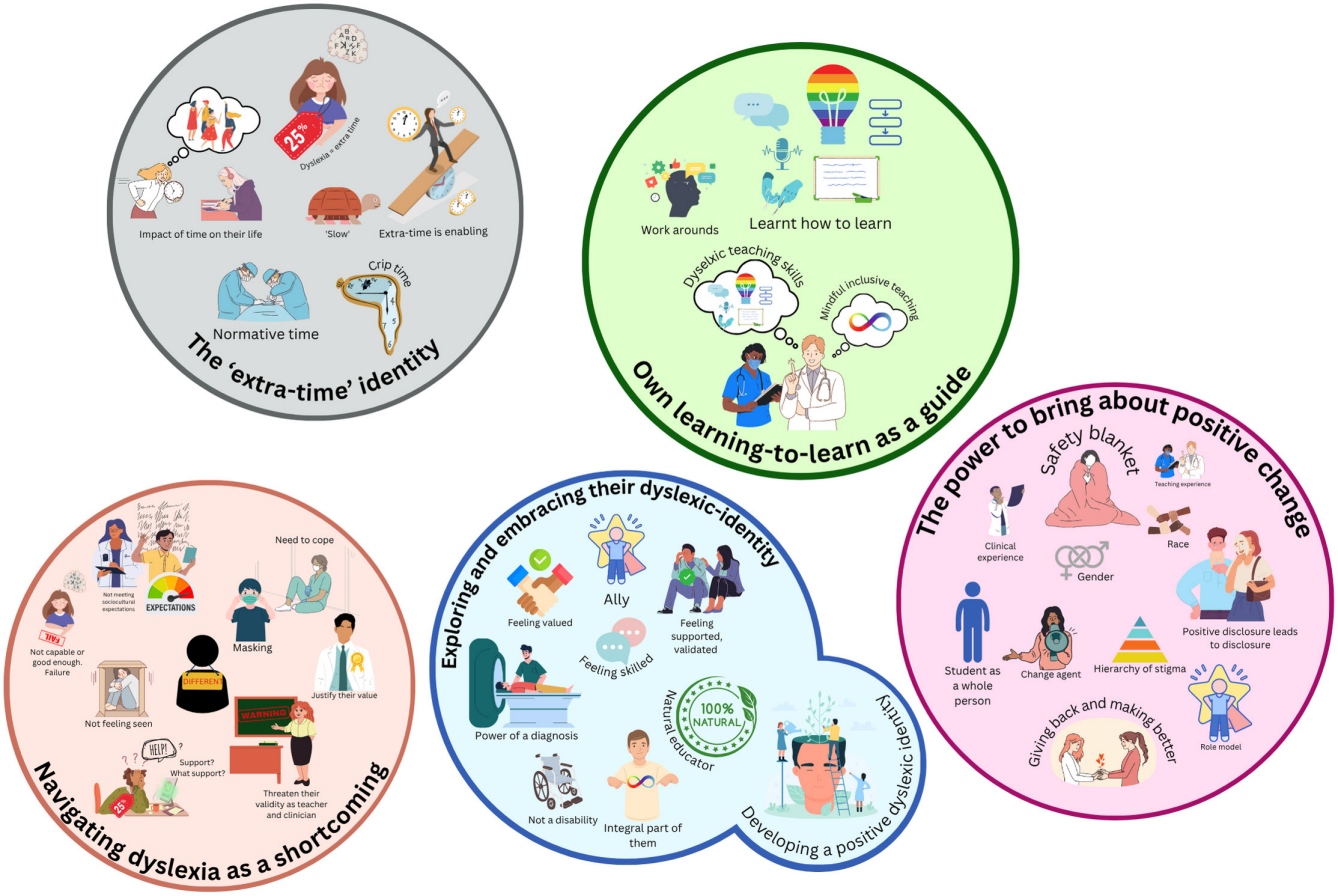
Development of themes.

Through reflexive thematic analysis, five themes were developed.

### 4.1 Theme 1: Navigating Dyslexia as a Shortcoming

Participants described falling short of perceived norms in medicine and education, such as an absence of mistakes.

You don't want to go she's done the operation, but she can't bloody spell it. (Rebecca)

Other examples of perceived norms included rapid scribing, structured phone calls, writing on the board and using mnemonics. Participants perceived that others view dyslexia and intelligence as mutually exclusive, resulting in dismissal, invalidation and, in some cases, delayed diagnosis.

Support accessed during medical training was often standardised and suited to academic settings, with limited transferability into clinical environments.

I long since lost my yellow cellophane thing to go over the screen and I'm not sure it would help that much. (Daniel)

Participants navigated less supportive environments through multiple strategies, including ‘needing to cope’, justifying their value and ‘masking’ to avoid being discredited.

I will always make a laugh if I write something on the board and it's wrong. I will just say oops. (Katie)

To some degree, all participants experienced their dyslexia in a deficit discourse and had developed strategies to navigate clinical and educational environments.

### 4.2 Theme 2: The ‘Extra‐Time’ Identity

The participants and Cleone had both used extra time in examinations as a reasonable adjustment since diagnosis.

They described times when their diagnosis was distilled to an ‘extra‐time’ label.

It basically was, do you want extra‐time or not? (Jane)

They felt pressure to conform to ‘normal time’.

I've got no time between operations because I'm sitting going … I can't spell anastomosis. (Rebecca)

Compensation strategies were used to recuperate extra‐time. Daniel explained, ‘I'm a much slower reader and writer, my thought processes are really quick … that actually averages out pretty quick’.

Participants took extra time to prepare teaching for student benefit. However, they recognised this was at personal expense.

I'm gonna put 10‐hour worth of work into that one‐hour lecture … I have to prepare quite extensively … This is when I do good teaching. (Arthur)

Though a need for extra preparation time was identified, participants seemed unaware of other supports that could assist their teaching.

[I] haven't accessed anything, I haven't really needed anything to do the teaching job, there's nothing that I can think of. (Katie)

### 4.3 Theme 3: Exploring and Embracing Their Dyslexic Identity

Participants recalled their dyslexia diagnosis as a powerful and meaningful event, bringing clarity to their day‐to‐day experiences. Understanding and embracing their dyslexic identity took time.

it was a bit reassuring, … it's not just me, it's not a lack, it's an actual thing. (Rebecca)

Participants recognised their dyslexia as a part of themselves:

I'm dyslexic and I'm a surgeon. These are both things that [are] part of my identity … to be perfectly frank, it's not a disability in my eyes anyway … I just do things differently. (Arthur)

Role models and allies enabled participants to positively explore their dyslexic identity.

One of the trainees stood up, called [the consultant] out and walked out the room, said they thought [calling a dyslexic person slow] was disgraceful. (Daniel)

Participants identified skills they brought to their profession, creating a sense of value and belonging, thereby strengthening their dyslexic identity. They portrayed themselves as instinctive educators:

[dyslexia] kind of drew me to teaching … I knew how to explain things in a simple way, friends, peers, all kind of gravitated to me. (Daniel)

### 4.4 Theme 4: Own Learning‐to‐Learn as a Guide

Participants shared that traditional learning methods were ineffective for them as students, so they developed their own learning strategies.

I went through the syllabus and literally every night was drawing and taking pictures … that's how I learn. (Arthur)

They then shared these strategies when teaching:

[the student said] ‘I'm dyslexic so I will never understand the words’ … [I said] so am I, so I've always learned it as colours and then just put the words next to it … then she said she completely understood it. (Katie)

*I've always learned it as colours and then just put the words next to it … then she said she completely understood it*.

Participants expressed learning‐style assumptions [22] when explaining how they adapted their approach for different learners.

I try to use a lot of different approaches … work for kinesthetic learners, something for reflective learners. Yeah, some people prefer didactic teaching. [I] use lots of components. (Daniel)

Participants adapted the learning environment to suit their own teaching preferences and perceived this benefitted their learners too.

I find it really beneficial if we're talking about equipment to just get it out and get them to handle it … I find it much easier, and students that I've taught always seem to find that very beneficial. (Jane)

Participants recognised diversity in the ways students learn and flexibly implemented a range of accessible, visible and practical teaching strategies.

### 4.5 Theme 5: The Power to Bring About Positive Change

Dyslexic medical educators were motivated and conscientious teachers, wanting to give back positive experiences and remedy negative ones.

I've wished I'd had something, so I've gone and set it up. (Jane)

Participants perceived they held power and responsibilities to be role models, advocates and challenge stigma.

Making sure that people aren't dismissed as not being able to do something because they don't do things the way that it's expected. (Rebecca)

*Making sure that people aren't dismissed as not being able to do something because they don't do things the way that it's expected*.

They identified that sharing their dyslexic identity positively with students created safe, supportive environments. In several examples, positive disclosure led to further disclosure.

I'm saying that I'm dyslexic and then two or three people in the group coming up to me [to say, ‘me too’]. (Jane)

Cleone reflected that she felt more comfortable to disclose to students whom she had met recurrently or spent longer with, suggesting trust may promote disclosure.

Participants felt a duty to disclose but noted that aspects of their identity, such as their gender and race, acted as ‘safety blankets’ protecting their credibility and enabling safe disclosure.

I would never want other people to have to come out … if I can do it to take the load off them, then that's good. (Daniel)

Participants reflected on dyslexia's position on a hierarchy of stigma and as part of an intersectional identity.

I think dyslexia is … becoming something of normality. But I think if you said, someone was neurodivergent or someone was autistic. I don't know if that would be as accepted. (Arthur)

## 5 Discussion

This research aimed to understand how dyslexic medical educators perceive being dyslexic influences their teaching practices and what motivates dyslexic doctors to teach. Participants in this study told a common story of self‐development, from navigating dyslexia as a shortcoming to becoming more comfortable and adopting a position of role model and ally, particularly in their teaching roles. We have therefore adopted this chronology in discussing the findings and to champion neurodiversity‐affirming approaches moving forward.

### 5.1 Navigating a Deficit Lens

Participants' experiences of failure, dismissal, invalidation, misconceptions and reductionist views suggest that in medicine, dyslexia continues to be viewed through a deficit lens [12]. Narrow clinical and educational norms equate intelligence with cognitive speed, so dyslexia risks being simplified to ‘slowness’ and a label of ‘extra time’ [13, 23, 24]. This is of concern, as repeated inferences about dyslexic ability and identity can lead to masking and overcompensating, negatively impacting a person's sense of self and belonging [10, 13, 23, 25]. This highlights the importance of medical educators identifying and championing their own and others' strengths and preferences.

### 5.2 Harnessing Challenges and Realising Strengths

Experiences of exclusion motivated participants to counter negative perceptions of dyslexia and prevent similar experiences for others. Diagnosis played a role in legitimising past experiences and helped them identify their dyslexic strengths. Allies encouraged participants to embrace their dyslexic identities by offering acceptance and proactive support and by challenging unwelcoming others. Previous authors have recommended dyslexia awareness training, allyship models and inclusive workplaces [12, 26, 27, 28]. We agree that supportive relationships and inclusive environments are vital, including for those without formal support, awaiting diagnosis, self‐identifying, undiagnosed or unable to disclose.

When dyslexic medical educators embraced their own dyslexic identities, they could openly and positively disclose being dyslexic to their students, and they became effective role models. As successful educators, their dyslexia was perceived positively, which reassured medical students. Disclosure led to an environment of openness, inspiring others to disclose and enabling educators to tailor support for their students. This finding underscores the importance of role modelling and visibility as crucial forms of support. However, participants acknowledged that parts of their identity (‘safety blankets’) facilitated safe disclosure. Not all dyslexic medical educators may feel safe to disclose, nor should feel pressured to do so. Disclosure may be context dependent, and developing inclusive environments remains crucial.

Limited success with traditional learning approaches, such as attending lectures and note‐taking, necessitated alternative learning strategies. Participants explored and embraced diverse learning methods, which improved their understanding and retention of information, and led to academic and teaching success. They encouraged students to explore different learning strategies, leveraged creativity to simplify complex topics and incorporated a range of techniques such as visualisation and hands‐on engagement to present content accessibly. They considered their strong communication and lateral thinking skills as assets and reflected on the meaningful connections they had created with students.

Dyslexic medical educators in our study enjoyed teaching and viewed it as an affirming aspect of their dyslexic identity. Teaching provided a platform to convey their strengths, positively demonstrating and reinforcing their contribution to their medical profession. Consistent with dyslexic teachers [16, 17], participants described a sense of purpose and personal achievement in overcoming barriers. Satisfaction in helping students and sharing learning techniques sustained and motivated them, and they were rewarded through bringing about positive change.

### 5.3 Recommendations

We propose the following recommendations for support and growth of dyslexic individuals in health professions education (Table 1). Firstly, increase visibility and inclusion of diversity, including dyslexia, in healthcare by fostering environments where individuals can embrace their authentic, intersectional identities. Challenge disabling environments to allow differences to manifest as strengths. Allies and role models are essential. There is a need for expanded allyship and intersectional mentorship training at all levels, and a meaningful shift towards inclusive language in policy documents [3, 26, 27].

**TABLE 1:** Table of recommendations.

Recommendations for supporting a dyslexia‐affirming approach in health professions education
1 Contribute to a safe and inclusive workplace by recognising and valuing diverse abilities and perspectives
2 Apply universal design principles to create accessible learning experiences for all
3 Include exploration and evaluation of learning strategies as explicit learning within health professions curricula
4 Participate in coaching and/or mentoring to continue developing yourself and others
5 Expand allyship and intersectional mentorship training within institutions at all levels
6 Update policy documentation to inclusive, non‐deficit‐based language

Secondly, we recommend shifting ‘learning how to learn’ from the hidden curriculum [29] towards an explicit, guided programme from the start of medical studies so that all students can confidently develop, implement and evaluate their own learning strategies.

Thirdly, improve individualised support for dyslexic professionals within healthcare. Current provisions are often inadequate, rigid and poorly translate to clinical environments [13, 24]. We advocate empowering individuals through coaching, mentoring and meaningful dialogue to support them in building positive identities and recognising their valuable contributions and skills.

### 5.4 Limitations

Our research was limited by a relatively homogenous participant group and intersectionality could have been explored further. By necessitating a formal diagnosis of dyslexia, we excluded self‐identifying individuals and those unable to access or able to disclose their diagnosis, aligning with a medical model, rather than the neurodiversity paradigm that became the frame for this research. Due to the time constraints of a Masters project and the importance of participant safety and trust, we chose to recruit participants already known to Cleone, rather than removing the influence of existing relationships. As a result, we did not explore the experiences of dyslexic doctors who do not teach or who find it hard to share their diagnosis. A larger study may be possible to gather a more diverse range of perspectives on dyslexia and teaching.

## 6 Conclusion

We found that dyslexic medical educators continue to face barriers created by a deficit discourse but challenge these by embracing their dyslexic identity and strengths as educators. Support should be targeted at both developing individuals and creating inclusive environments. When diversity is visible and valued, dyslexic medical educators can act as role models and allies using their insights to create inclusive learning environments and support students. All medical students should be provided with opportunities to explore and evaluate a variety of learning strategies, which may help avoid failure and reduce stigma. We recommend adopting a neurodiversity‐affirmative approach and championing the role of dyslexic medical educators in developing more inclusive health professions education.

## Author Contributions

**Cleone Riad:** conceptualisation, methodology, data curation, investigation, resources, validation, writing – original draft, visualisation, project administration, formal analysis, writing – review and editing. **Alison Ledger:** formal analysis, writing – review and editing, supervision, validation, methodology.

## Funding

The authors have nothing to report.

## Ethics Statement

Ethical approval has been granted by the University of Leeds School of Medicine ethics committee (Ref.: MREC 22‐005).

## Consent

No patients were included in this study. Participants gave informed consent for participation and publication of this research.

## Conflicts of Interest

The authors declare no conflicts of interest.

## Data Availability

Research data (original transcripts) are not shared due to the sensitive nature of the topic and potential for participant identification.
